# Grounding Causal Closure or Something Near Enough

**DOI:** 10.1007/s12136-024-00620-4

**Published:** 2024-12-28

**Authors:** Bradford Saad

**Affiliations:** Global Priorities Institute, University of Oxford, Oxford, UK

**Keywords:** Mental causation, Causal closure, Dualism, Physicalism, The mind-body problem, The exclusion problem, Overdetermination

## Abstract

A causal argument for physicalism is widely held to pose a problem for dualism. This view has an unobvious presupposition, namely that the causal closure of the physical has a special sort of ground. The requisite sort of ground must distinguish the causal argument for physicalism from many defective causal arguments. On behalf of physicalists, I develop an account of the ground for the causal closure of the physical, thereby putting the causal argument for physicalism back in the business of causally problematizing dualism. One consequence of my account is that physicalists can pose a causal problem for dualism using a much weaker closure premise than is generally assumed.

## Introduction

Physicalists and dualists agree that physical events exist. But whereas physicalists claim that every event is physical, dualists claim that nonphysical mental events exist too. It is widely thought that a causal argument poses a problem for dualism.^1^ However, that argument has a largely unnoticed presupposition, namely that the causal closure of the physical has a special sort of ground: a ground that distinguishes the causal argument for physicalism from many sound-yet-defective causal arguments against entities (for example, unicorns) that would be efficacious if they existed. As we will see, supplying such a ground is not simply a matter of providing reasons to accept the causal closure of the physical domain. And there is reason to think that supplying such a ground would compromise the causal argument by generating an objectionable form of overdetermination.^2^ The task of this paper is to develop an account of the ground of the causal closure of the physical, or something near enough, that enables physicalists to defend the causal argument.

Here is the plan: Section 2 rehearses the causal argument. Section 3 unpacks the noted objection to that argument and describes the strategy for responding to that objection that I will develop on behalf of physicalists. Section 4 preempts a worry about that strategy. Section 5 implements the strategy by examining modifications of the causal argument that are designed to defend it against the objection in question and recommending one such modification. Section 6 considers how dualists might respond to the modified causal argument. Section 7 shows how physicalists can block those responses. Section 8 takes stock.

## The Causal Argument

To get the main issues on the table, let us start with a simple formulation of *the causal argument*:

**Closure**: Every physical effect has a physical cause.

**Mental Efficacy**: Every mental event causes a physical event.

**No Overdetermination**: Physical events with physical causes lack nonphysical causes.

Therefore, there are no nonphysical mental events—i.e., all mental events are physical.

I will call physicalists who take the causal argument to pose a causal problem for dualism *causal argument proponents*.^3^ Some comments and clarifications are in order.

First, I opt for a simple formulation because incorporating bells and whistles from more sophisticated formulations would distract from the issues I most wish to discuss. For example, I omit references to times. And I construe No Overdetermination as a universal ban on the overdetermination of physical events via physical causes and nonphysical causes. Invoking a weaker ban that merely prohibits systematic overdetermination would yield a more plausible argument, but incorporating that ban and adjusting the other premises accordingly to preserve validity would needlessly complicate the discussion.

Second, I remain neutral between the standard construals of “physical,” *modulo* the following stipulation: the physical domain is closed under realization. It follows from this stipulation that nonphysical events are not realized by physical events; this obviates the need for cumbersome talk of mental events to which dualists are committed as nonphysical events that are not realized by physical events. This stipulation also ensures that *non-reductive physicalists*—who posit mental events that are realized by events that are, in a standard sense, physical—will qualify as physicalists on the adopted definition of physicalism.

Third, it might be wondered: why is Mental Efficacy formulated as a universal claim rather than as the standard and more plausible existential premise that some mental events cause physical effects? The answer is that the increased plausibility of that premise in standard formulations is purchased at the price of invalidity. Given Closure and No Overdetermination, what follows from that existential claim is that some mental events—namely the ones that cause physical events—are physical. But that result is compatible with the existence of other mental events that are nonphysical, and hence with the truth of dualism and the falsity of physicalism.^4^ Alternatively, one could opt for a non-standard formulation of the argument that retains the existential premise and restores validity by adding a bridge premise either saying that if some mental events cause physical effects, then all do, or else saying that if some mental events are physical, then all are. Recasting my discussion in terms of the resulting formulations would affect little substance. However, adopting a formulation with a bridge premise would complicate the discussion. So, for simplicity, I adopt the above formulation of the argument rather than one of these other valid formulations.^5^

Fourth, I understand causation as a relation that holds between events. While I think that the causal argument is best run with a productive, “oomphy” notion of causation, I will remain neutral on that controversial issue throughout this discussion. Whether causal relations invoked in the causal argument should be understood as sufficient causal relations rather than causal relations of a weaker sort will be taken up in due course. Although Closure is typically understood in terms of sufficient causation, one upshot of my discussion will be that causal argument proponents have reason to adopt a weaker version of Closure.

Finally, I flag that structurally similar causal arguments have been advanced in support of reductive physicalism, dualism, physicalism about color, mereological nihilism, the identity of dispositions with categorical bases, and the view that composition is identity; such arguments have also been wielded against role functionalism and non-naturalism about morality.^6^ In fact, I believe that much of what I say about the dialectic between physicalists and dualists can be plausibly extended to debates concerning some of these positions and their rivals. However, arguing for that exceeds the scope of the present paper.

## A Dilemma for Causal Argument Proponents

Before unpacking the charge that causal argument proponents cannot supply a ground of the right sort for Closure’s truth, it will help to first illustrate why not just any sort of ground would do. Following Tiehen (2015a, 2015b), we can illustrate that by examining a defective causal argument against unicorns. Call an event a *unicorn event* if it has a unicorn as its constituent object and a *non-unicorn event* otherwise. The causal argument against unicorns proceeds as follows:

**Closure**_**U**_: Every physical effect has a non-unicorn cause.

**Unicorn Efficacy**: Every unicorn event causes a physical event.

**No Overdetermination**_**U**_: No unicorn event overdetermines physical effects—i.e., physical effects with non-unicorn causes lack unicorn causes.

Therefore, there are no unicorn events and hence no unicorns.

Despite being sound and causal, this argument reveals nothing *causally* problematic about unicorns. For Closure_U_ is true *only because* unicorns do not exist. Indeed, if there were unicorns, they would non-redundantly cause effects—for example, hoofprints—that violate Closure_U_. Thus, it is the non-existence of unicorns—not Closure_U_’s truth—that deprives unicorns of causal efficacy. Closure_U_’s truth does not have a ground of the sort it would need to engender such causal woes.^7^

On reflection, this argument against unicorns turns out to belong to a large class of defective causal arguments with true closure premises: every false existential claim entails a true closure thesis that can be used to underwrite a sound causal argument against that existential claim. But many false existential claims are causally unproblematic. I have no twin. There is no Bigfoot. There are no dodo birds. There is no highest natural number. It is not because the existence of these entities would pose causal problems that I deny their existence. I happily admit that my twin would have causal powers if (s)he existed, as would Bigfoot and dodo birds. And it is not for want of causal powers that the highest natural number fails to exist—other natural numbers manage just fine without such powers. Thus, the mere fact that there is a sound causal argument from a closure thesis against a view does not show that that view has a causal problem. What more is needed?

One tempting answer may be that a sound causal argument must supply a closure premise with appropriate epistemic credentials: that is, with evidence that provides warrant for accepting the premise.^8^ However, notice that we are warranted in accepting the causal closure of the non-unicorn domain. Yet this does not enable the causal argument against unicorns to show that unicorns are causally problematic—as we have seen, that argument shows no such thing. Likewise, providing warrant for accepting Closure is not by itself enough to enable the causal argument to problematize dualism. Since the causal argument is supposed to pose a metaphysical problem for dualism, such epistemic credentials would have to trace to metaphysical considerations.^9^ Thus, answers of this sort will be incomplete, at best.^10^

Another tempting answer may be that the truth of the closure premise needs to be modally robust, holding across all possible worlds. Unlike the previous answer, this one at least offers metaphysical credentials. Unfortunately, the credentials license defective causal arguments. For the supposition that unicorns are metaphysically impossible,^11^ this answer entails that the causal argument against unicorns poses a causal problem for the existence of unicorns. But, even on that supposition, there is nothing causally problematic about unicorns.

Tiehen’s (2015a: §6) discussion gestures at the following better answer: the *right sort of ground* of a closure premise in a causal argument aimed at posing a causal problem for a view *X* is a ground^12^ that would have ensured the closure premise’s truth even if *X* had been true. I will assume that this answer is correct.^13^ So, where *X* = dualism, a ground of the right sort is one that would have ensured Closure’s truth even if dualism were true. Thus, I contend that unless Closure’s truth has such a ground, the putative causal problem for dualism is a myth.

To make matters worse for causal argument proponents, Tiehen (2015b) argues that positing a ground of the requisite sort for Closure’s truth would undermine the causal argument’s ban on overdetermination. This line of reasoning, to be elaborated in Section 4, underwrites a dilemma for causal argument proponents: if Closure’s truth lacks a ground of the right sort, then the causal argument for physicalism suffers the same defect as the above argument against unicorns. But if the causal argument for physicalism has such a ground, then No Overdetermination is undermined. Either way, the causal argument fails to causally problematize dualism.

As a physicalist, Tiehen responds to this dilemma by rejecting the causal argument and conceding that dualism is causally unproblematic. As a dualist, I would welcome the discovery that Tiehen’s dilemma exonerates dualism from its alleged causal problem. But, on behalf of physicalists, I advocate a different response to the dilemma: since I agree that the right sort of ground is unlikely to be forthcoming for the truth of standard versions of Closure, I advise causal argument proponents to use a non-standard version of Closure, a version that is significantly weaker than standard versions and unlike standard versions admits of a ground of the right sort. As a segue to developing this response, I will consider and allay the worry (alluded to above) that this sort of response would undermine the causal argument.

## Groundwork for an Un-Pyrrhic Victory

My response to the above dilemma grasps its first horn: I aim to vindicate the causal argument’s ability to causally problematize dualism by offering a ground of the right sort for the truth of a version of Closure. To do this, I need to overcome an objection that awaits on this horn, threatening to render Pyrrhic the victory of any causal argument proponent who supplies a ground of the right sort. The objection, recall, is that positing such a ground would undermine Non-Overdetermination.

To elaborate, the objection can be understood as resting on two premises. The first premise is that, by positing a ground of the right sort for Closure’s truth, causal argument proponents incur a commitment to the overdetermination of Closure’s truth by distinct grounds.^14^ Why accept this premise? Well, causal argument proponents are by definition physicalists. And the truth of physicalism would explain Closure’s truth by ruling out the only type of entity that could violate Closure, namely nonphysical events. But physicalism’s truth does not cause Closure to be true. So, causal argument proponents should regard physicalism’s truth as a ground of Closure’s truth. Further, since physicalism would not have been true if dualism were true, physicalism’s truth would not have grounded Closure’s truth if dualism were true. It follows that physicalism’s truth is not a ground of the right sort for Closure’s truth. Thus, upon supplying a ground of the right sort for Closure’s truth, causal argument proponents would have to posit at least two grounds for Closure’s truth: physicalism’s truth and a distinct ground of the right sort—this is the commitment to overdetermination asserted by the first premise.

The second premise is that such overdetermination would be akin in relevant respects to causal overdetermination of the sort banned by the causal argument. Why accept the second premise? After all, whereas No Overdetermination bans *causal* overdetermination, the causal argument proponent who posits the right sort of ground for Closure’s truth countenances *non-causal* (*grounding*) overdetermination. In response, the objector will claim that this is a distinction that makes no difference. For causal overdetermination is problematic in virtue of involving redundant determination—a feature it shares with non-causal overdetermination—not because it involves causation *per se*.^15^ Proponents of causal arguments sometimes admit as much.^16^

From these premises, the objector concludes that, upon positing the right sort of ground for Closure’s truth, causal argument proponents undermine No Overdetermination and hence the causal argument. I will now answer the objection by offering a ground of the right sort for Closure’s truth—on one reading of Closure—and contending that no problematic overdetermination ensues, even supposing that physicalism is true. To be clear, my aim at this juncture is to answer the above objection, not to restore the causal argument’s ability to causally problematize dualism.

Here, then, is a ground of the right sort:

- **(S1)**: Every physical event is in spacetime,^17^ and
- **(S2)**: Every event in spacetime with a cause has a physical cause.

Call this conjunction (*S&*). (S&) entails Closure. (S1) is a conceptual truth on some construals of “physical.” Even on readings of “physical” on which (S1) is not a conceptual truth, (S1) is an extremely plausible claim that even dualists should accept. Perhaps some dualists—for example, sense-data theorists—may wish to posit a realm of mental events outside of spacetime. But even such dualists lack a basis for positing a realm of *physical* events outside of spacetime. Thus, Occam’s razor counsels dualists to deny that such a realm exists.

(S2)’s plausibility will turn on what sort of causal notion is in play. Since our current aim is to provide a case of unproblematic non-causal overdetermination, let us opt for a maximally weak causal notion in order to maximize (S2)’s plausibility. In particular, let us understand (S2) in terms of causal influence, where an event *c causally influences* an event *e* just in case *c* makes at least some causal contribution—however small—to *e*.^18^ So understood, (S2) is extremely plausible: even on interactionist versions of dualism that forgo redundant mental causes, (S2) will presumably be true. If nothing else, (S2) will be true because the precise spatiotemporal character of any given event in spacetime that has a cause will be influenced by the distribution of matter.^19^

Having noted their plausibility, I hereafter assume that (S1) and (S2) are true, and hence that (S&) is true. Of course, our understanding of (S1) and (S2) must be reflected in the version of Closure we take them to entail. (I will continue to use “Closure” by itself in a way that is neutral as between different readings of it that causal argument proponents might adopt.) That version of Closure is:

**Influence Closure**: Every causally influenced physical event has a physical causal influencer.

(S&)’s truth evidently grounds Influence Closure’s truth: in addition to metaphysically necessitating Influence Closure’s truth, (S&)’s truth provides a plausible, non-causal explanation of why Influence Closure is true. A point in favor of this explanation is that it lends to a plausible, deeper explanation of Influence Closure’s truth in terms of fundamental laws. For it is plausible that: fundamental dynamical laws and fundamental laws that constrain the boundary conditions ground (S1)’s truth,^20^ fundamental dynamical laws ground (S2)’s truth, and these grounds together explain Influence Closure’s truth by explaining (S&)’s truth. One reason this deeper explanation is appealing is that fundamental laws are good candidates for unexplained explainers.

Notice that Influence Closure’s truth would also be grounded in physicalism’s truth: while physicalism’s truth does not cause Influence Closure’s truth, it does explain Influence Closure’s truth by ruling out the only possible counterexamples to Influence Closure, namely nonphysical causal influencers. On the supposition that physicalism is true, should we, then, conclude that Influence Closure’s truth is problematically overdetermined by physicalism’s truth and (S&)’s truth? Intuitively, the answer is “no.” Witness the fact that this overdetermination of Influence Closure’s truth does not bear any of the hallmarks of problematic overdetermination: that physicalism’s truth and (S&)’s truth each explain Influence Closure’s does not involve an unexpected convergence in explanatory powers (compare: a priori, there is no reason to expect convergence in causal powers between nonphysical mental events and their physical bases); nor is there is suspicious additivity violation; nor is how the overdetermined phenomenon occurs oddly insensitive to variations in the overdeterminers.^21^ Thus, we have a truth (Influence Closure) that causal argument proponents should regard as non-causally and unproblematically overdetermined.

Where does this leave the causal argument proponent with respect to the above objection? Well, that objection used an analogy between causal and non-causal overdetermination to establish a presumption in favor of regarding as problematic the overdetermination of Closure’s truth by physicalism’s truth and a ground of the right sort. But the causal argument proponent now has a case in which a thesis that is at least in the vicinity of Closure (namely Influence Closure) is true and unproblematically overdetermined by physicalism’s truth and a ground of the right sort. That case is, I contend, enough to defeat the noted presumption, and hence to disarm the objection.

With the objection disarmed, causal argument proponents no longer have reason to think that finding the right sort of ground for Closure’s truth undermines No Overdetermination, and hence the causal argument’s ability to causally problematize dualism. Thus, having deflected a threat of Pyrrhic victory, causal argument proponents may now undertake in earnest the tasks of finding the right sort of ground for Closure’s truth and using that ground to rehabilitate their argument.

## How to Ground the Causal Argument Without Defanging It

I argued above that, on the supposition that physicalism is true, Influence Closure’s truth is unproblematically overdetermined by physicalism’s truth and a ground of the right sort, namely (S&)’s truth. That result was used to remove an obstacle from the path of causal argument proponents. With the obstacle removed, they have two options. First, they might discard (S&) or Influence Closure and seek some other way of supplying the causal argument with a rightly grounded closure premise. A second, simpler option runs the causal argument using Influence Closure as a premise and (S&)’s truth as a ground of the right sort. Let us try the second option.

### Vindication by Substitution

Here is a natural suggestion for implementing that option: after advancing (S&)’s truth as a ground of the right sort for Influence Closure’s truth, use Influence Closure as a premise and substitute causal influence for causation throughout the argument, yielding:

**Influence Closure**: Every causally influenced physical event has a physical causal influencer.

**Mental Influence**: Every mental event causally influences a physical event.

**No Influential Overdetermination**: Physical events with physical causal influencers lack nonphysical causal influencers.

Therefore, all mental events are physical events.

Unfortunately, this does not do the trick. The trouble is that No Influential Overdetermination issues a ban that is far stronger and correspondingly less plausible than No Overdetermination, on standard readings of that premise. For No Overdetermination is typically read as targeting a form of *redundant* mental-physical causation. In contrast, No Influential Overdetermination bans forms of redundant and *joint* causation (i.e., the non-redundant causation of an effect by multiple causes). But joint causation is ubiquitous and intuitively unproblematic. As a result, No Influential Overdetermination is inapt to problematize dualism.^22^

### Vindication by Sufficiency?

The upshot of Section 5.1 was that no argument that relies on No Influential Overdetermination will causally problematize dualism. This is unlikely to come as a surprise. For no overdetermination premises in causal arguments are often formulated as bans on overdetermination via *sufficient* causes.^23^ But this suggests a way forward: find out what kind of Closure premise such arguments need, and then find the right sort of ground for the truth of such a premise. To that end, consider:

**Sufficient Closure**: Every physical event with a sufficient cause has a sufficient physical cause.

**Mental Sufficiency**: Every mental event causally suffices for a physical event.

**No Sufficient Overdetermination**: Physical events with sufficient physical causes lack sufficient nonphysical causes.

Therefore, all mental events are physical events.

On the above suggestion, the next step for causal argument proponents is to find the right sort of ground for Sufficient Closure. As it happens, most causal argument proponents accept Sufficient Closure, at least if objective probability-fixing causes qualify as sufficient.^24^ Perhaps, there is a ground of the right sort for Sufficient Closure in the vicinity of (S&)’s truth. The natural candidate is (S&)_SUF_, understood as the conjunction of:

- **(S1)**: Every physical event is in spacetime, and
- **(S2)**_**SUF**_: Every event in spacetime with a *sufficient* cause has a *sufficient* physical cause.

(S1) remains plausible. Moreover, (S2)_SUF_ is obviously true on the supposition that physicalism is true. However, recall, what is needed is a ground for Sufficient Closure that would ground Sufficient Closure’s truth even if dualism were true. (S&)_SUF_ meets that condition only if (S2)_SUF_ would have been true if dualism were true. But it is far from clear that (S2)_SUF_ would have been true if dualism were true.

This can be appreciated by attending to the difference between the influence reading of (S2) (on which every event in spacetime with a causal influencer has a physical causal influencer) and (S2)_SUF_. The influence reading of (S2) is plausible because we think of spacetime as saturated with chains of physical causal influencers.^25^ Given this picture, it is hard to see how a physical event could inhabit spacetime without falling along one of these chains. This picture of spacetime is robust under various suppositions about mentality. Even dualists who countenance mental causes that are neither physical nor redundant are under pressure to accept it. That is why the influence reading of (S2) would remain true even if dualism were true.

In contrast, (S2)_SUF_ ensures that every physical event with a cause has a physical causal guarantor. This *automatically* renders redundant any contribution to physical reality by a nonphysical event. Thus, to assert that (S2)_SUF_ would be true even if dualism were true is to assume that nonphysical mental events would be redundant or epiphenomenal, i.e., that dualism has a causal problem. But that would beg the question in the present context.^26^

Perhaps causal argument proponents can avail themselves of some other ground for Sufficient Closure’s truth that is of the right sort or else cultivate a non-question begging case for (S2)_SUF_ and, in turn, (S&)_SUF_. But there is a more promising track that I will explore instead.

### A New Hope

I noted above that the causal argument is often run with a premise like:

**No Sufficient Overdetermination**: Physical events with sufficient physical causes lack sufficient nonphysical causes.

This premise has the virtue of exhibiting plausibility not shared by its counterpart No Influential Overdetermination. However, it is not without sin: taken as a premise, it puts the causal argument proponent in need of a ground of the right sort for Sufficient Closure’s truth, a ground that has not been forthcoming. But there is an alternative. Some authors instead run the argument with a premise like:^27^

**No Redundancy**: Physical events with sufficient causes lack distinct causal influencers.

No Redundancy captures the motivation behind No Sufficient Overdetermination—it precludes physical effects of mental causes from being redundantly caused—but without committing No Influential Overdetermination’s sin of overreach in its exclusion of unobjectionable joint causation.

The standard way of running the causal argument with a premise like No Redundancy goes something like this:^28^

**Sufficient Closure**: Every physical event with a sufficient cause has a sufficient physical cause.

**Mental Influence**: Every mental event causally influences a physical event.

**No Redundancy**: Physical events with sufficient causes lack distinct causal influencers.

Therefore, all mental events are physical events.

Running the argument in this way would not constitute an advance for causal argument proponents. For they will still need to supply a ground of the right sort for Sufficient Closure’s truth. But there is another way of running the causal argument with No Redundancy.

### No Redundancy Meets Influence Closure

Recall that we abandoned an attempt to run the causal argument with Influence Closure that required an implausibly strong ban on overdetermination. And we abandoned an attempt to run the causal argument with a plausible ban on overdetermination (No Sufficient Overdetermination) because it required a closure premise (Sufficient Closure) for which we failed to find a ground of the right sort. Properly employed, No Redundancy yields a causal argument that displays the virtues of these attempts while avoiding their vices. One such argument is:

**Influence Closure**: Every causally influenced physical event has a physical causal influencer.

**Mental Sufficiency**: Every mental event causally suffices for a physical event.

**No Redundancy**: Physical events with sufficient causes lack distinct causal influencers.

Therefore, all mental events are physical events.

Call this the *causal argument from Influence Closure*. Whereas the previous No Redundancy-invoking argument construed mental events as influencers to put them into competition with sufficient physical causes, this argument reverses the causal statuses of mental and physical competitors: it construes mental events as sufficient causes to put them in competition with physical influencers.

The causal argument from Influence Closure causally problematizes dualism. For Influence Closure is a truth with the right sort of ground, namely (S&)’s truth. Premises like Mental Sufficiency and No Redundancy appear in standard iterations of the causal argument. Being accompanied by Influence Closure rather than a stronger more familiar closure premise does not diminish their plausibility. In any event, No Redundancy is intuitively plausible. And Mental Sufficiency cannot be rejected without enfeebling the causal status of the mental.

## A Dualist Attempt at Damage Control

Dualists cannot escape the foregoing causal argument without paying a theoretical price. But the price of escape remains up for negotiation. Two lines of escape are familiar from dualist responses to standard causal arguments that appeal to Sufficient Closure. The first countenances mental-physical overdetermination,^29^ which here manifests as a denial of No Redundancy. Anyone who regards positing such overdetermination as an adequate dualist response to standard causal arguments is likely to regard it as an adequate response to the causal argument from Influence Closure. On the second line, dualists opt to live with epiphenomenalism (which denies that mental events have physical effects).^30^ I think that these are hard lines that dualists need not take in response to standard causal arguments, given that a ground of the right sort has not been supplied for their closure premises. But I admit that dualists who take these lines do not have to pay any *additional* cost to escape the causal argument from Influence Closure.

Having noted these responses, I will set them aside in order to examine the prospects for a new interactionist dualist response that opens up when causal argument proponents relinquish standard causal arguments in favor of the causal argument from Influence Closure. The response is as follows. The causal argument from Influence Closure problematizes only extremely robust versions of interactionist dualism that construe *all* nonphysical mental events as *sufficient* causes. Even slightly less robust forms of interactionist dualism remain unscathed. One such view asserts that every mental event *almost* (though not quite) causally suffices for a physical event. Another maintains that mental events *systematically* causally suffice for physical effects, while allowing that mental events occasionally lack the causal strength required to fulfill Mental Sufficiency’s universal ambitions.

At least initially, this response seems promising as a dualist means of damage control: it concedes that the causal argument from Influence Closure generates problems within the ranks of dualist positions, but maintains that the problems afflict only a limited subset of dualist views. And it does so without taking on any theoretical commitments as costly as epiphenomenalism or the postulation of universal mental-physical overdetermination. In the next section, I introduce three ways to resist this response on behalf of causal argument proponents. The upshot will be that, despite its initial promise, the noted response does not provide an easy dualist escape from causal woe.

## How Causal Argument Proponents Might Respond

Causal argument proponents have a number of options for resisting the above dualist attempt at damage control. I will outline what I regard as the most promising options.

### First Response

Admittedly, Mental Sufficiency may seem unduly strong when we consider the characteristic physical effects of mental events. For example, although I take my intention to type these words to cause them, I readily admit that my intention is not sufficient on its own: were it not for my fingers, my heart, and my keyboard, my intention would have no such effects. Thus, I do not take my intention to type these words to be a *sufficient* cause of them. This line of thought suggests that dualists can easily give up Mental Sufficiency and hence that the proposed way of running the causal argument inflicts little damage on dualism.

While tempting, the above line of thought is misguided. Its fault lies in its exclusive focus on the *characteristic* physical effects of mental causes. Such effects are by and large behavioral. As such, they are plausibly *mediate*, rather than *immediate*, effects of mental causes. Mental causes bring about such effects only by intermingling with physical causes. And it is a familiar fact about causal connections that they tend to weaken with mediation.^31^ Whereas a sufficient cause c of an effect e must hold that status over intervening links in the causal chain between c and e, c need not—and often does not—retain that status over e’s progeny. It is no wonder, then, that mental events are not always sufficient causes of their characteristic physical effects. No parallel line of reasoning suggests that mental events are not sufficient causes for their immediate effects. But absent reason to doubt that mental events causally suffice for their immediate effects, there is no reason to doubt Mental Sufficiency.

Moreover, when we shift our attention from characteristic physical effects of mental causes to the vast array of candidate physical effects, we encounter support for Mental Sufficiency. For one, Mental Sufficiency could be made true by mental events qualifying as sufficient causes of events intrinsic to arbitrarily small regions of spacetime. For another, Mental Sufficiency could be made true by mental events causally sufficing for *probabilistic* physical events.^32^^,^ 33

Suppose it is granted that mental events are not sufficient causes of their characteristic physical effects. And—for the moment—bracket the causal argument from Influence Closure. Question: do dualists then have any reason to deny that mental events are sufficient causes of non-characteristic physical effects, such as probabilistic events in arbitrarily small regions? Evidently, the answer is “no.” If so, dualist should assign some credence to the dualist hypothesis that nonphysical mental events are sufficient causes of such effects. Once the causal argument is unbracketed and dualists learn that it rules out this view, they should reduce their confidence in dualism.

### Second Response

Mental Sufficiency requires *all* mental events to be sufficient causes of physical events. If this requirement is met, the only way for mental events to non-redundantly cause physical events without violating Influence Closure is for mental events to be physical events. A dualist intent on damage control can allow that that requirement is strictly unmet due to the occasional causally enfeebled mental event, while holding that it is systematically met and so met near enough.

Here, I advise causal argument proponents to grant that Mental Sufficiency imposes a requirement that is merely systematically met and adjust the causal argument from Influence Closure to problematize the resulting dualist position. The adjustment starts by replacing Mental Sufficiency with the requirement that mental events systematically causally suffice for physical effects. For example, causal argument proponents might concede that not every hope, dream, and glimmer of phenomenology causally suffices for a physical effect, while insisting that most total conscious experiences in normal humans do. The next adjustment weakens No Redundancy’s universal ban on overdetermination via sufficient causes and causal influencers to a ban on systematic overdetermination of that sort. Given Influence Closure, these adjustments deliver systematic mental-physical identities. To reach the desired conclusion—that every mental event is physical—causal argument proponents need only invoke the (plausible) premise that every mental event is physical if systematic mental-physical identities are obtained.

### Third Response

The dualist maneuver under consideration contends that the causal argument from Influence Closure rules out a limit case (in which all mental events causally suffice for physical events) without threatening the range of dualist views for which that case is a limit. Causal argument proponents can respond in kind.

Here is a sketch of how this option works. Its main contention is that the causal argument from Influence Closure is a limited case for a range of causal arguments, a significant portion of which causally problematizes dualism. The arguments in the range are ordered by the (logical) strength of their closure premises, with Influence Closure being the weakest closure premise of any argument in the range.^34^ Transitioning from the argument from Influence Closure to causal arguments with stronger closure premises comes at a cost: all else equal, the stronger the closure premise, the harder it will be to supply the premise’s truth with a ground of the right sort. This is a cost because a causal argument causally problematizes dualism only if the truth of its closure premise has a ground of the right sort. The argument from Sufficient Closure in Section 5.2 occupies some point in this range. By that point, closure premises have been strengthened so much that it is doubtful that grounds of the right sort for them can be found. In light of this, the best candidates for additional causal arguments that causally problematize dualism will fall in the range between the causal arguments from Influence Closure and causal arguments from Sufficient Closure.

A net loss in plausibility need not result from the cost incurred in the transition from the causal argument from Influence Closure to a causal argument with a stronger closure premise. For replacing Influence Closure with a stronger closure premise affords the opportunity to weaken the mental causation and no overdetermination premises. The gains from such weakening may exceed the cost of strengthening the closure premise.

As advertised, this is only a sketch of this option for thwarting the dualist’s attempt at damage control. To fill it in, the causal argument proponent would need to:

1. Supply intermediate causal notions (between causal influence and sufficiency).
2. Use those notions to formulate intermediate closure premises.
3. Argue that the truth of the resulting premises have grounds of the right sort, and
4. Adjust (presumably by weakening) the remaining premises in the causal argument.

Defending an implementation of (1)–(4) is a research project that exceeds the scope of this paper. But here are a few suggestions for undertaking that project.

Regarding (1), the causal argument proponent would do well to start with intermediate causal notions already in use. Some candidates are as follows: counterfactual dependence causation, probability raising causation, difference-making causation, *de facto* dependence causation, and proportionate causation.^35^ Given an intermediate causal notion i, executing (2) will simply be a matter of plugging i into the following schema:

**Schematic Closure**: Every physical event with an *i*-cause has a physical *i*-cause.

To argue in accordance with (3) that true instances of this schema have the right sort of ground, causal argument proponents may appeal to instances of:

**Schematic (S&)**: Every physical event is in spacetime and every event in spacetime with a cause has a physical *i*-cause.

There is no guarantee in advance that instances of Schematic (S&) that appeal to a stronger causal relation than causal influence will provide grounds of the right sort for their associated closure premises. Causal argument proponents will have to assess such instances on a case-by-case basis. Still, the fact that the substitution instance that uses causal influence constitutes a ground of the right sort for its associated closure premise encourages optimism here.

Two strategies for implementing (4) deserve mention. Suppose causal argument proponents secure some intermediate causal notion i and the right sort of ground for its associated closure premise. Then, the first strategy for implementing (4) replaces No Redundancy with a weaker premise in the following argument:

**i-Closure**: Every physical event with an i-cause has a physical i-cause.

**Mental Sufficiency**: Every mental event causally suffices for a physical event.

**No i-Redundancy**: Physical events with sufficient causes lack distinct i-causes.

Therefore, all mental events are physical events.

Suppose again that causal argument proponents secure some intermediate causal notion i and the right sort of ground for i-Closure. Then, the second strategy implements (4) by adopting the following argument:

**i-Closure**: Every physical event with an i-cause has a physical i-cause.

Mental i*-Efficacy: Every mental event i*-causes a physical event, where an i* is an intermediate causal relation (whether i = i* is left open).

No i/i*-Redundancy: Physical events with i* causes lack distinct i-causes.

Therefore, all mental events are physical events.

This argument differs from both the previous argument and the argument from Influence Closure in two respects. First, this argument replaces Mental Sufficiency with a weaker premise, namely Mental i*-Efficacy. Second, it replaces No Redundancy with a premise that is stronger in one respect and weaker in another: whereas No Redundancy induces causal competition between maximally strong and maximally weak causes (i.e., sufficient causes and causal influencers), No i/i*-Redundancy induces a competition between pairs of intermediate causes (possibly of different sorts).

## Conclusion

Sound yet defective causal arguments abound. Many such arguments fail to causally problematize their targets because their closure premises are true simply in virtue of their targets’ non-existence. Should physicalists admit this fate for the causal argument? That depends on whether they can find a ground of the right sort for the causal closure of the physical domain, i.e., a ground that would have sustained the causal closure of the physical even if dualism were true. For all anyone has previously argued, physicalists may be unable to supply a ground of the right sort for standard closure premises (which appeal to sufficient physical causes). However, there is at least one closure truth—Influence Closure—that enjoys the right sort of ground, and there is reason for optimism about the existence of other such truths (Section 7.3). Influence Closure is much weaker, logically speaking, than those standardly used in the causal argument for physicalism. It merely requires physical effects to have physical causal influencers. Given the universal (as far as I know) use of stronger closure premises in causal arguments, I suspect that many causal argument proponents would deny that Influence Closure captures, in a sense in which they are interested, the causal closure of the physical. Yet, in addition to having the right sort of ground, Influence Closure turns out to be strong enough to causally problematize dualism. Thus, proponents of the causal argument can rest assured that the causal closure of the physical—or something near enough—is on firm ground.

## Footnotes

1 For various formulations of the argument, see Bennett (2007: 357), Chalmers (2015: 251), Gibb (2015: 627), Kim (2011: 214-7), Ney (2016: 145), Papineau (2002: Ch. 1), Tiehen (2015b: 2411), Tye (2009: §2.3), and Yablo (2009: 223-4).

2 This objection to the causal argument is due to Tiehen (2015a, 2015b). Related worries about using closure premises to support physicalism or to tell against dualism have been raised, often in passing, by Bennett (2008: 282-3), Bishop (2006), BonJour (2010), Buhler (2020), Chalmers (2010: Ch. 5, fn24, fn26), Dimitrijević (2020), Garcia (2014), Gibb (2015), Koons and Bealer (2010: xix), Lowe (2000: 572), Montero (2003), Ney (2016), Sturgeon (1998), Tiehen (2022); cf. Zhong (2020). In what follows, I just focus on Tiehen’s objection. I take his objection to be one of the more developed and promising objections of this sort and also to be underexplored. But for some discussion of it, see Bikaraan-Behesht (forthcoming), Dimitrijević (2020), Di Francesco and Tomasetta (2015), Ney (2022), and Saad (2017, 2018).

3 Strictly speaking, the argument does not yield physicalism, as its conclusion is compatible with the existence of nonphysical events that are non-mental. But since such events play no role in the dialectic between dualists and physicalists, the argument in effect entails physicalism in this context.

4 Kim (2005) defends such a dualist view according to which some mental events are causally efficacious and physical while others are epiphenomenal and nonphysical.

5 Thanks to reviewers for pressing me to elaborate here.

6 Respectively, see Kim (1998), Jackson (1998: Ch. 4), Saad (2018), Merricks (2001), Wallace (2011), Heil (2005), Kim (2010: Ch. 10), and Bedke (2009).

7 N.B. The point here is *not* to argue that a causal argument against nonphysical mental events is in trouble because it is *exactly* parallel to a defective argument against unicorns. Rather, the unicorn argument serves to illustrate how a causal argument can be defective in virtue of its true closure premise lacking a ground of the right sort. This, in turn, supports the claim that causal argument proponents cannot settle for just any sort of ground for Closure’s truth.

8 For an influential empirical case for closure, see Papineau (2002: Appendix).

9 For an extended defense of this point, see Tiehen (2015a: §3). I take a metaphysical problem for a view to concern what the view claims about reality, not our ability to know or justify what the view claims. Thus, I do not assume any connection between a priori accessibility and a problem’s being metaphysical. Thanks to a reviewer for comments that prompted these clarifications.

10 This is not to say that evidence that has been offered in support of Closure is irrelevant to physicalism. Even evidence for Closure that does not by itself enable the causal argument to problematize dualism might do so when combined with other considerations. There is also the possibility of enlisting evidence that has been offered for Closure (e.g., by Papineau) to underwrite a *non-causal* argument for physicalism. For a suggestion along these lines, see Tiehen (2015b: §8).

11 Cf. Kripke (2011: 65).

12 Some truth A grounds a truth B just in case A non-causally and metaphysically explains B. I assume that grounds metaphysically necessitate whatever they ground and that the explanatory grounding relation between grounds and what they ground is asymmetric and transitive. I remain neutral on whether grounding can be analyzed in more basic terms. Those who take grounding to be non-transitive can read my grounding claims in terms of the ancestral of grounding. Those who prefer to think of grounding as a sentential operator rather than a relation are invited to recast the discussion in terms of their preferred notion. Thanks to a reviewer for prompting these clarifications.

13 One might object that this answer delivers the wrong result in the case of the highest natural number. After all, if there were a highest natural number, the relevant closure claim would have remained true. Yet, intuitively, there is nothing *causally* problematic about the hypothesis that there is a highest natural number. My response is that while the relevant closure claim has a ground of the right sort, the relevant efficacy claim (that every highest-natural-number event causes a physical event) does not, as it is vacuously true because no highest natural number exists and would be false if one did. Although this reveals that sound causal arguments can be defective despite having closure premises with grounds of the right sort, in what follows, I will focus on whether there is a ground of the right sort for the closure premise. Whether causal argument proponents can provide Mental Efficacy with a ground of the right sort is a companion challenge whose development lies beyond the scope of this paper.

14 N.B. the premise allows that some of these grounds may not be of the right sort.

15 See Tiehen (2015b: 2412) and Saad (2020). For an account of grounding that exploits the analogy between causation and grounding, see Schaffer (2016).

16 E.g., see Kim (1995: 239; 2005: 17-21).

17 Supposing spacetime as conceived in our best current physical theories will be succeeded by a more fundamental physical arena—perhaps of a sort envisioned in quantum gravity or string theory—we can here understand “spacetime” broadly to encompass that arena.

18 Cf. Lewis (2000).

19 See, e.g., Maudlin (2012: Ch. 6).

20 Or else (S1)’s truth is grounded in what it is to be a physical event.

21 See Block (1990: 159), Bernstein (2016: 30-2), and Paul and Hall (2013: 148–149, 157–158).

22 Admittedly, No Influential Overdetermination only bans joint causation by physical and nonphysical causal influencers, and proponents of the causal argument deny that such joint causation exists. However, what is supposed to be problematic about positing nonphysical causes in the context of the causal argument is their redundant character, not their nonphysicality. Thus, the causal argument’s non-overdetermination premise should rest on a more-general, theory-neutral non-overdetermination principle—a principle that would be plausible even if dualism were true. The ubiquity of unproblematic joint causation in the physical domain indicates that no plausible principle of that sort is available for No Influential Overdetermination and hence that No Influential Overdetermination cannot serve to problematize dualism.

23 E.g., see Árnadóttir and Crane (2013: 253), Bennett (2008: 281), and Kim (2005: 42). I prefer to understand sufficient causation in a strong manner that entails nomic necessitation (or the objective probability-fixing) of the effect by the cause. But some authors operate with weaker notions of sufficient causation on which sufficient causes of effects necessitate those effects *given certain contextually determined background conditions*. Such weaker notions probably have a closer relationship with causal talk. However, I think their imprecision and contextual variability inhibit metaphysical theorizing. For example, I find no overdetermination premises formulated in terms of them difficult to assess. That said, I invite those who prefer a weaker notion to read what follows in terms of their preferred notion. For discussion of how different notions of causal sufficiency bear on the exclusion problem, see Bennett (2003).

24 See Gibb (2015: 629).

25 For example, in special relativity, we may regard any physical event in spacetime as a causal influencer of the events in its future light cone and as causally influenced by all physical events in its past light cone.

26 Put differently, if we are to accept (S2)_SUF_ as part of a ground (S&)_SUF_ of the right sort for Sufficient Closure’s truth, then we need some reason for thinking that (S2)_SUF_ is not true merely because physicalism is true.

27 E.g., see Chalmers (2015: 251), Yablo (2009: 223), and Gibb (2015: 627).

28 See *ibid*.

29 See Chalmers (1996: 135-6), Kroedel (2015), List and Stoljar (2017), and Mills (1996).

30 Authors who at least sympathize with epiphenomenalist dualism include Chalmers (1996: Ch. 4-5; 2010: 130-2), Gertler (2019), Jackson (1982), and Robinson (2006).

31 There may be exceptions. (Strong nuclear force interactions are perhaps the best candidates). If so, I suspect that they are scarcer than they might seem. For example, suppose that tomorrow we stumble across long lost works of Aristotle. After millennia of collecting dust, the works induce a frenzy of scholarly activity. Is this not a case in which a cause bears more responsibility for more mediated effects than less mediated ones? For would not Aristotle’s writing those works then enjoy more causal responsibility for effects tomorrow than for effects today? No. Having more causal responsibility for more-mediated effects is one thing. Having causal responsibility for more—as in a greater number—of mediated effects is another. Causes in the first category would yield exceptions to the attenuation of causal strength with mediation, but Aristotle’s writing the noted works plausibly is not in that category. Aristotle’s writing the noted works plausibly belongs to the second category, but causes in that category need not yield exceptions to attenuation of causal strength with mediation—cf. Yablo (2009: 283).

32 Those who have scruples with probabilistic events may prefer this alternative: replace Mental Sufficiency with the claim that every mental event causally suffices to fix the probability of some physical event, and No Redundancy with the claim that physical events with probability-fixing causes lack distinct causal influencers.

33 Another suggestion in the vicinity: Mental Sufficiency could be made true by mental events qualifying as sufficient causes of extremely coarse-grained events. I advise against adopting this suggestion. All else equal, the more coarse-grained an event is, the more apt it is for *unproblematic* overdetermination; cf. Bunzl (1979). Thus, by trafficking in coarse-grained events, causal argument proponents tempt dualists to adopt an overdetermination-countenancing response. Causal argument proponents would do well to block this dualist response by restricting the effects mentioned in the causal argument to fine-grained events.

34 The ordering may not be strict, as the stronger closure theses may exceed Influence Closure in strength along different dimensions that are not logically comparable.

35 See Lewis (1973), Lewis (1986), List and Menzies (2009: §II), Yablo (2010: Ch. 4), and Yablo (2009: 283).
